# “We're All in the Same Boat”: A Review of the Benefits of Dragon Boat Racing for Women Living with Breast Cancer

**DOI:** 10.1155/2012/167651

**Published:** 2012-06-28

**Authors:** Susan R. Harris

**Affiliations:** Department of Physical Therapy, Faculty of Medicine, University of British Columbia, 212-2177 Wesbrook Mall, Vancouver, BC, Canada V6T 1Z3

## Abstract

This narrative review summarizes findings from quantitative and qualitative research literature that has been published over the past 15 years since an initial, community-based pilot study first challenged the long-held medical belief that vigorous, upper-body exercise would lead to lymphedema in women who were at risk due to treatments for breast cancer. Dragon boat racing originated in China more than 2000 years ago and has become a popular recreational and competitive support around the world. From the advent of the world's first breast cancer survivor dragon boat team, *Abreast in a Boat* launched in Vancouver, British Columbia, in 1996, there are now more than 140 breast cancer survivor dragon boat teams paddling and competing in 12 different countries. The wealth of quantitative and qualitative research that has ensued since that pilot study further supports the initial hypothesis that resistance exercise, for example, dragon boat paddling, is not only *safe* for women recovering from conventional breast cancer therapies but also shows that dragon boating has been embraced as a complementary exercise therapy by the cancer survivors participating in this magical sport.

## 1. Introduction

Dragon boat racing originated in China more than 2000 years ago and is steeped in culture and powerful rituals. Traditionally, dragon boats raced from the north of China, the region of death, to the south—the realm of life [1]. Hong Kong hosted the first international dragon boat festival in 1976, and, 10 years later, the sport came to North America as part of Expo 86 in Vancouver, British Columbia. According to Barker: “Hosting dragon boat races is thought to bring health, happiness and prosperity, as well as offer protection for the unfriendly spirits of the sea” [1].

In 1996, Vancouver hosted the first international dragon boat festival held outside China. Among more than 130 teams entered from around the world was a novice team comprised entirely of women who had been treated for breast cancer, aptly named *Abreast in a Boat*—to signify not only the 24 paddlers' seated positions within the large, 700-kg boat but also the fact that many of them each had only “*a* breast.” The brainchild of Dr. Donald McKenzie, a sports medicine physician and exercise physiologist at the University of British Columbia [2], the goal of *Abreast in a Boat* that first year was to determine whether women at risk for upper extremity lymphedema could progressively train for and partake in this repetitive, resistive sport without developing lymphedema—the chronic, irreversible swelling of the arm for which they were at greatly increased risk [3].

The purpose of this narrative review is to summarize the research, both quantitative and qualitative, that has ensued in the 16 years since *Abreast in a Boat* first took to the water in Vancouver. The quantitative research will trace the trajectory from the initial pilot study that examined whether or not this vigorous, repetitive upper-body sport was safe for women at risk for lymphedema through to recent, more rigorous studies, for instance, randomized controlled trials and systematic reviews, which have continued to explore the safety and benefits of resistive exercise for breast cancer survivors. The qualitative research to be summarized explores the meanings of participating in this sport to some of the several thousand women around the world who are living with breast cancer and who have paddled and competed in dragon boat racing since those initial 24 pioneers first raised their paddles in Vancouver.

## 2. Potential Risks of Vigorous, Repetitive Upper-Body Exercise

For years prior to the launch of *Abreast in a Boat*, rehabilitation and oncology health professionals had warned women who had undergone axillary dissection for the staging of breast cancer to avoid strenuous, repetitive upper-body activity under the long-held presumption that such type of exercise would lead to development of lymphedema, a chronic and irreversible swelling of a limb [4–7]. This longstanding belief was based on the fact that exercise increases blood flow and would thereby increase lymph production, possibly leading to lymphedema [8]. Although removal of axillary lymph nodes and axillary radiation are known risks for developing lymphedema in the affected extremity, there was no evidence that exercise would actually induce lymphedema in those at risk or exacerbate preexisting lymphedema, and yet this myth has continued to prevail.

In a recent survey of 175 Australian women who had been treated for breast cancer, 70% reported that they intended to avoid strenuous activity with the involved upper extremity, due to “fear of lymphedema” [9]. 

The first study to challenge this longstanding belief was a case series involving upper-extremity measurements that was conducted on 20 of the 24 paddlers in *Abreast in a Boat* during their first paddling season in 1996. Results of that small pilot study have been supported by a number of subsequent studies involving larger samples and more rigorous research designs. These studies will be described and summarized in the following section.

## 3. Quantitative Research on Upper-Body Exercise for Women with Breast Cancer

### 3.1. The Pilot Dragon Boat Study

Although *Abreast in a Boat* began primarily as a community recreational activity to enable women living with breast cancer to reclaim their formerly healthy selves, two physical therapists—one a team member and the other a team coach—quantitatively studied the effects of resistance training and paddling on the risk of developing lymphedema by taking serial measurements of the paddlers' arm circumferences at four standardized anatomical locations at three time points: prior to paddling training, 2 months after commencement of training, and 7 months after the race season [10]. There were no clinically significant interlimb circumferential differences at the final time-point [10].

These results led the authors to conclude that women who had undergone axillary dissection and, in almost two-thirds of cases in the sample, radiation to the breast and/or axilla may be able to partake safely in strenuous upper body exercise. In a commentary accompanying the published article, a renowned surgical oncologist remarked that the study “should serve as the impetus for a more formalized randomized trial to confirm the authors' hypothesis, and again put a surgical myth to the test of a scientific study” [11]. Fortunately, that is exactly what happened in the decade following publication of this landmark initial study.

### 3.2. Other Quantitative Studies on Dragon Boat Racing

Whereas the initial pilot study [10] took place entirely within a community setting, the first sub-sequent dragon boat study was laboratory-based and more rigorously controlled, albeit also a case-series design [12]. Lane and colleagues examined the effects of a 20-week resistance and aerobic exercise program, supplemented at week 8 with addition of dragon boat training, for 16 breast cancer survivors [12]. Upper-extremity circumference and volume and upper-body strength were assessed at baseline, week 8 and week 20. In contrast to findings in the pilot study [10], all outcome measures increased significantly between times 1 and 3 for women in the combined exercise program [12]. However, as Lane et al. concluded, changes were consistent across both upper extremities and the increases in arm volume were likely due to the accompanying strength changes and not to lymphedema.

In 2009, McNeely et al. studied the effects of an acute bout of moderate-intensity exercise on upper-extremity volume in 23 breast cancer survivors regularly participating in dragon boat racing [13]. Each participant had undergone unilateral axillary lymph node dissection or sentinel lymph node biopsy, with 17 also having had radiation treatments. Five of the 23 participants had preexisting lymphedema. The independent variable was 20 minutes of continuous exercise on an arm ergometer. Although limb volume increased bilaterally immediately following the exercise bout, it reduced to levels slightly below baseline 60 minutes after exercise completion. The study authors concluded that moderate-intensity bouts of exercise are safe for breast cancer survivors with and without lymphedema but that their findings could be generalized only to women who had been participating already in vigorous upper-body exercise, that is, dragon boat racing.

Unfortunately, all of the research to date that has explicitly involved dragon boat racing as an intervention (or dragon boat paddlers as participants) has employed case-series designs with small samples [10, 12, 13]. However, this early line of inquiry that challenged the longstanding belief that vigorous, repetitive exercise was unsafe for women at risk for lymphedema [10] led to a number of larger and more rigorous studies examining the effects of other types of upper-body resistance training on lymphedema risk and other outcome variables.

### 3.3. Other Research on Upper-Body Resistance Training

Over the past 5 years, at least seven systematic reviews (SRs) have been published examining the effects of resistance training and other forms of exercise on survivors of breast and other types of cancer [14–21]. The two most relevant to the focus of the current article are a 2008 SR by Cheema and colleagues, summarizing the effects of progressive resistance training on health-related benefits and potential adverse effects, for instance, lymphedema, in women with breast cancer [14], and a 2011 review by Kwan et al. examining the safety of resistance exercise for breast cancer patients with or at risk for lymphedema [15].

In their SR of 10 studies published between 1966 and 2007, Cheema et al. found no incidence or exacerbation of quantified or self-reported lymphedema as a result of resistance exercises or a combination of resistance and aerobic exercise [14]. In the more recent SR, Kwan and colleagues included 17 studies or SRs (2004–2010) of resistance and/or aerobic exercise training as well as other interventions, for instance, shoulder range of motion and manual lymph training; the authors of this SR concluded that slowly progressive resistance exercise is safe at any time following breast cancer surgery [15]. Both SRs reported improvements in muscle strength as a result of resistance training [14, 15].

Although the studies included in these two SRs [14, 15] were not limited to those involving resistance exercises used in dragon boat racing, their results support the findings from the original *Abreast in a Boat* case series [10], that is, that vigorous, repetitive upper body exercise does not initiate lymphedema or exacerbate preexisting lymphedema. Given that the studies reviewed included 11 randomized controlled trials (RCTs), many with samples much larger than that in the original pilot study, there is reasonable substantiation that resistance exercise or programs involving a combination of aerobic and resistance exercise are safe for women who have had axillary lymph nodes removed and, in many cases, radiation to the breast and/or axilla. The fact that three of the studies included in the most recent SR [15] were well-powered RCTs [22–24] provides further compelling evidence for the safety and efficacy of resistance exercises in women with pre-existing lymphedema [22] or those at risk for the disorder [23, 24].

### 3.4. Summary

Since the 2000 publication of the first small study to challenge the belief that vigorous upper-body exercise could lead to lymphedema, an impressive body of quantitative research on similar types of resistance exercise has continued to support the original hypothesis “that women who have undergone axillary dissection and, in many cases, radiation for the treatment of breast cancer *may *be able to safely engage in strenuous, repetitive upper body exercise” [10]. This subsequent line of research has shown also that resistance exercise, either alone or in combination with aerobic exercise, has positive benefits on muscle strength, body composition, self-esteem, and most importantly the participants' quality of life [14, 15, 21].

## 4. Qualitative Research on the Experience of Dragon Boat Racing

Since *Abreast in a Boat* first took to the water in 1996, dragon boat racing for women with breast cancer has become a worldwide phenomenon. According to the *Abreast in a Boat* website [25], there are now 143 breast cancer dragon boat teams in 12 different countries. With approximately 25 paddlers per team, these figures suggest that more than 3500 breast cancer survivors are enjoying the mysticism, camaraderie, and overall good fun that this magical sport engenders.

Because there is much more to dragon boat racing than getting fit and challenging long-held myths about the dangers of vigorous sport, a number of qualitative researchers have chosen to study this powerful phenomenon by exploring what the sport means to the paddlers themselves [26–31]. Published between 2002 and 2011, these six studies involving 67 women resulted in a number of themes, several of which were remarkably similar: feelings of camaraderie, a sense of renewed fitness and health, opportunities to promote awareness of a full and enjoyable life after breast cancer, and enhanced self-confidence and control of one's life.

Feelings of *camaraderie* were echoed by a number of participants, as seen in the following quote:

**“The feeling of racing, giving it your all using every ounce of muscle that you possibly have. That feeling of strength, the camaraderie. Getting us all together*. You can do it if there's 22 people in the boat, you can go anywhere. It's everybody, all working paddling the same...*” [26, page 53]. **

Within the predominant theme of physical and emotional well-being, one woman described her *renewed feelings of self-confidence, fitness, and taking back control of her life* [27]:

*...That's why I think dragon boating is so important to women because it starts to build their confidence back that they can do something, taking control of their lives and puts them in fantastic physical shape...through all of the accomplishments you have a feeling of control back and of confidence back. Then you start feeling great from a physical aspect...comes the confidence and from the confidence comes the control, it's all weaved together *[27, page 143]. **

In another study [30], a participant described how the experience of dragon boat racing gave her a sense of *renewed health*:

*I found [dragon boat racing] actually improved my physical condition. I used to have very severe osteoporosis, and I had lower back pain, and when I started paddling, because you use your whole body and you use your lower back, I was worried that it would cause too much strain on my back and it would be difficult, but it had the opposite effect. After a while, my back pain actually went away, so it was really beneficial *[30, page 229]. **

Although dragon boat racing has not been included in lists of more standard complementary therapies for cancer, it clearly fits within definitions provided by leading experts in that area in that it serves as an “adjunct to mainstream cancer care” [32, page 80] and is aimed at enhancing well-being (and controlling symptoms) of its participants [32]. As can be seen from both the quantitative and qualitative studies summarized in the foregoing two sections, involvement in this sport has enhanced the well-being of its participants and assisted in controlling their symptoms.

Not only has physical exercise been shown to contribute to increasing muscle strength and enhancing overall fitness and well-being, it has been shown also to improve overall quality of life and to prolong survival in women who have been diagnosed with breast cancer.

## 5. Benefits of Physical Activity on Quality of Life and Survival after Breast Cancer

Two recent meta-analyses have summarized the benefits of physical activity/exercise interventions on health-related quality of life (HRQoL) [33] and mortality after a breast cancer diagnosis [34]. Duijts and colleagues reported a summary effect size of 0.298 (**P* < 0.001*) based on 13 studies examining the effect of physical exercise on HRQoL [33], thus providing quantitative support for some of the participants' quotes in the preceding section that summarized qualitative research findings for dragon boat racing. Other statistically significant effects of physical exercise interventions were reported in decreasing fatigue and depression and enhancing body image.

In their meta-analysis of six studies on physical activity and survival after breast cancer diagnosis, Ibrahim and Al-Homaidh found that postdiagnosis physical activity reduced breast cancer deaths by 34%, all cause mortality by 41%, and breast recurrence by 24%; however, when women with estrogen-receptor-negative breast cancer were analyzed separately, there were no significant effects for that sub-group which comprises about one-quarter of women with the disease [34]. The authors surmised that the greater benefits of physical activity for women with estrogen-receptor-positive disease were likely due to the effects on reducing estrogen levels.

## 6. Discussion

Although the quantitative and qualitative studies included in this paper had different aims, their combined findings support dragon boat racing as a beneficial complementary therapy for women who have experienced breast cancer, regardless of whether or not they have lymphedema. Results of the quantitative research, both on dragon boating specifically as an intervention [10, 12, 13] and on other types of resistance exercises [14–24], suggest that these recreational pursuits are safe for individuals who have undergone axillary surgery and/or radiation to the breast, chest wall, or axilla and do not lead to development of lymphedema or exacerbation of pre-existing lymphedema. In addition, resistance exercise positively influences muscle strength, body composition, self-esteem and quality of life. Improvement in self-esteem and quality of life certainly fits with the aim of complementary therapies for cancer, that is, to enhance participants' well-being [32]. In their 2009 systematic review on the effects of exercise on quality of life in women with breast cancer, Bicego and colleagues included nine RCTs of moderate to high methodological quality and concluded that there was “strong evidence that exercise positively influences QOL in women living with breast cancer” [21, page 45]. 

Although exercise had not previously been considered as a *typical *complementary therapy for cancer, the 2009 clinical practice guidelines developed by the Society for Integrative Oncology included among their 20 overall recommendations a specific recommendation on exercise and physical activity: “Regular physical activities can play many positive roles in cancer care. Patients should be referred to a qualified exercise specialist for guidelines on physical activity to promote basic health.” [35, page 96]. The authors commented further that the strongest evidence for this recommendation was for breast cancer survivors and that resistance exercise is particularly beneficial during adjuvant cancer therapy [35]. 

Following completion of cancer treatments, the practice guideline authors recommend standard public health guidelines for cancer survivors [36], that is, to exercise at least 20–30 minutes at moderate-to-vigorous intensity on at least 3 to 5 days of every week. Because training regimens for dragon boat racing typically include both aerobic and resistive exercises, each of 20–30 minutes duration on 3 to 5 days of the week [10], this is an ideal recreational program for breast cancer survivors to maintain and/or regain post-treatment fitness. Furthermore, a recent systematic review has shown that exercise helps to mitigate the effects of cancer-related fatigue among breast cancer survivors [37], thus enhancing quality of life. 

Whereas quantitative research showed that dragon boating is *safe *for women who have undergone treatments for breast cancer, and enhances a number of physical and psychosocial outcomes, findings from qualitative research describe the tremendous joy, support, and camaraderie that this sport engenders [26–31]. Although qualitative research does not aim at generalizability, per se, most of the studies took place in Canada and involved primarily Caucasian, well-educated women. 

Despite this limitation, quantitative studies have shown that exercise and physical activity are complementary therapies commonly used by breast cancer survivors of different ethnic origins. Based on a questionnaire administered to over 5,000 Chinese women with breast cancer, Chen et al. reported that physical activity was the third most common complementary therapy used, with walking the most popular type of physical activity [38]. In a study of 125 Hispanic women living in the southwestern USA (with educational level averaging less than 10 years and income less that $20,000  per year), Owens and colleagues reported that 65% used exercise as a complementary therapy during breast cancer treatment [39]. Furthermore, in a South Korean survey of 425 breast cancer survivors, of the 57.4% that reported use of complementary/alternative medicine, exercise therapy was the most common type used by 43.2% [40]. Certainly the fact that there are breast cancer survivor dragon boat teams in Malaysia, Shanghai, and Singapore suggests that paddlers are not all Caucasian [25], although this is the ethnic group most heavily represented in the qualitative studies conducted to date.

## 7. Conclusion

Beginning with the “fledgling research efforts of a group of Canadian investigators” [41, page 710], a longstanding medical myth that had threatened to negatively influence the quality of life in breast cancer survivors was first challenged in Vancouver in 1996. The published, community-based pilot study from that initial research [10] spawned a host of subsequent and far more rigorous quantitative studies, including RCTs and systematic reviews that went on to support the initial hypothesis that vigorous, repetitive upper-body exercise was safe for women who had been treated for breast cancer. In addition, that original case-series led to a half-dozen qualitative studies that further supported the effects of dragon boating in enhancing participants' quality of life. 

Because exercise and physical activity have recently emerged as *mainstream* complementary therapies, the compelling line of research supporting the safety and positive effects of dragon boat racing and other forms of resistance exercise suggests that these are *evidence-based* complementary treatments. The universal joy of participating in dragon boating, as witnessed by participant quotes from the qualitative studies included in this paper, lends further support to the importance of making all women who have been treated for breast cancer aware of this wonderful recreational opportunity:

**“*When I am in a dragon boat, when I am dragon boating, I feel free, exhilarated, (pause) in control, powerful, all those good things.*” [28, page 133]. **
